# Pulmonary bacteriophage and cystic fibrosis airway mucus: friends or foes?

**DOI:** 10.3389/fmed.2023.1088494

**Published:** 2023-05-17

**Authors:** Kak-Ming Ling, Stephen Michael Stick, Anthony Kicic

**Affiliations:** ^1^Wal-Yan Respiratory Research Centre, Telethon Kids Institute, The University of Western Australia, Perth, WA, Australia; ^2^Occupation, Environment and Safety, School of Population Health, Curtin University, Perth, WA, Australia; ^3^Division of Paediatrics, Medical School, The University of Western Australia, Perth, WA, Australia; ^4^Department of Respiratory and Sleep Medicine, Perth Children's Hospital, Nedlands, WA, Australia; ^5^Centre for Cell Therapy and Regenerative Medicine, School of Medicine and Pharmacology, The University of Western Australia and Harry Perkins Institute of Medical Research, Perth, WA, Australia

**Keywords:** bacteriophage, cystic fibrosis, mucus, airway epithelium, antimicrobial resistance

## Abstract

For those born with cystic fibrosis (CF), hyper-concentrated mucus with a dysfunctional structure significantly impacts CF airways, providing a perfect environment for bacterial colonization and subsequent chronic infection. Early treatment with antibiotics limits the prevalence of bacterial pathogens but permanently alters the CF airway microenvironment, resulting in antibiotic resistance and other long-term consequences. With little investment into new traditional antibiotics, safe and effective alternative therapeutic options are urgently needed. One gathering significant traction is bacteriophage (phage) therapy. However, little is known about which phages are effective for respiratory infections, the dynamics involved between phage(s) and the host airway, and associated by-products, including mucus. Work utilizing gut cell models suggest that phages adhere to mucus components, reducing microbial colonization and providing non-host-derived immune protection. Thus, phages retained in the CF mucus layer result from the positive selection that enables them to remain in the mucus layer. Phages bind weakly to mucus components, slowing down the diffusion motion and increasing their chance of encountering bacterial species for subsequent infection. Adherence of phage to mucus could also facilitate phage enrichment and persistence within the microenvironment, resulting in a potent phage phenotype or vice versa. However, how the CF microenvironment responds to phage and impacts phage functionality remains unknown. This review discusses CF associated lung diseases, the impact of CF mucus, and chronic bacterial infection. It then discusses the therapeutic potential of phages, their dynamic relationship with mucus and whether this may enhance or hinder airway bacterial infections in CF.

## Introduction

Antimicrobial resistance (AMR) has a tremendous healthcare burden. It is estimated to account for over 10 million deaths (1) at an annual healthcare cost of $400 million in Australia alone by 2050 (2). Unfortunately, individuals with chronic conditions, including cystic fibrosis (CF), are likely to experience more severe consequences of AMR. Therapies such as antibiotics are essential for chronic bacterial eradication; however, their repeated and semi-continuous consumption is likely a key driver of AMR, especially in the CF population. Disappointingly, the discovery pipeline into the production of novel antibiotics is waning, and current innovation has not kept up with evolving resistance. The arrival of CFTR modulators has substantially improved the lung function of individuals with CF, but the benefit for inflammation and infection remains inconclusive (3). Therefore, there is still a great need for therapeutics that can complement CFTR modulators and reduce chronic bacterial infection.

Bacteriophage (phage) therapy has been proposed to tackle chronic bacterial infections in CF (4–6). Phages are found ubiquitously in the body and the natural environment and show strong potential for clinical use (7–9). Those applicable for therapy are typically lytic phages that recognize and bind to specific bacterial cell surface receptors (10, 11). Several clinical cases have demonstrated a reduction in bacterial density and clinical improvement when bacterial infections have been treated with phage (12–16). However, the full impact of phage therapy on pulmonary infection in CF is yet to be fully appreciated. Many unanswered questions include determining the best administration route, concomitant use of antibiotics or mucolytic agents, length of the treatment period, and phage formulation. Furthermore, additional basic research is needed to predict these parameters and accurately measure host immune responses.

In young children with CF, mucus flakes present very early in life (17) and are associated with inflammation and airway luminal hypoxia without bacterial infection and structural lung disease (18). The resultant mucus flakes create a microenvironment favorable for bacterial colonization (17, 19). The mucus layer is an essential entity facilitating phage diffusion across the mucosal surface, and recent evidence in the gut suggests that they can bind to parts of mucus, improving their bactericidal activity (11). Knowing that mucus and its structure in CF are distinctly unique, little is known about how this would impact phage functionality. In this review, we explore the clinical features of CF disease, antibiotic choice, and the underlying drivers of MDR. We summarize treatment options and provide evidence as to why phage therapy may be crucial for infection control in this population. We explore the feasibility and benefit of phage therapy in treating chronic infection in CF, including investigating phage behavior and efficacy in the CF lung microenvironment.

## Cystic fibrosis

### CF lung disease and what do we know about CF mucus?

Cystic Fibrosis is an autosomal recessive genetic disease that markedly impacts multiple mucosal surfaces, particularly the pancreatic ducts, intestinal mucosa and airway epithelium. The mutation of the cystic fibrosis transmembrane conductance regulator (CFTR) gene on chromosome 7 (7q31.2) results in defective chloride transport across the apical surface of the epithelial (20–22). Impaired ion transport, imbalanced water flow in the CF airway surface liquid volume, and airway dehydration prevent adequate cough clearance via the mucociliary escalator (23). There are also higher concentrations of mucins in the lungs of those with CF and elevated osmotic pressure of the mucus layers, which subsequently triggers thick, dense mucus production and drives muco-inflammatory airway obstruction (24–27). Recently, mucin content analysis in bronchoalveolar lavage fluid (BALf) of children with CF revealed both elevated mucin concentration and increased mucus burden typified by “mucus flakes” (17) which were associated with inflammation and airway luminal hypoxia (17, 18). The impact of dysfunctional mucus in CF on inhaled therapeutic agents such as antibiotics and chemical compounds has also been investigated (28, 29). Specifically, macromolecules (mucins and DNA) in CF mucus increased its viscosity and impeded the diffusion of therapeutic agents such as antibiotics (30). Furthermore, high salt concentrations and low oxygen levels have been shown to reduce antibiotic effectiveness (31, 32). Knowing this effect raises the question of whether the CF mucus could affect other treatment approaches, including phage.

### Chronic bacterial infections and associated treatment regimens in CF

**Figure 1:**
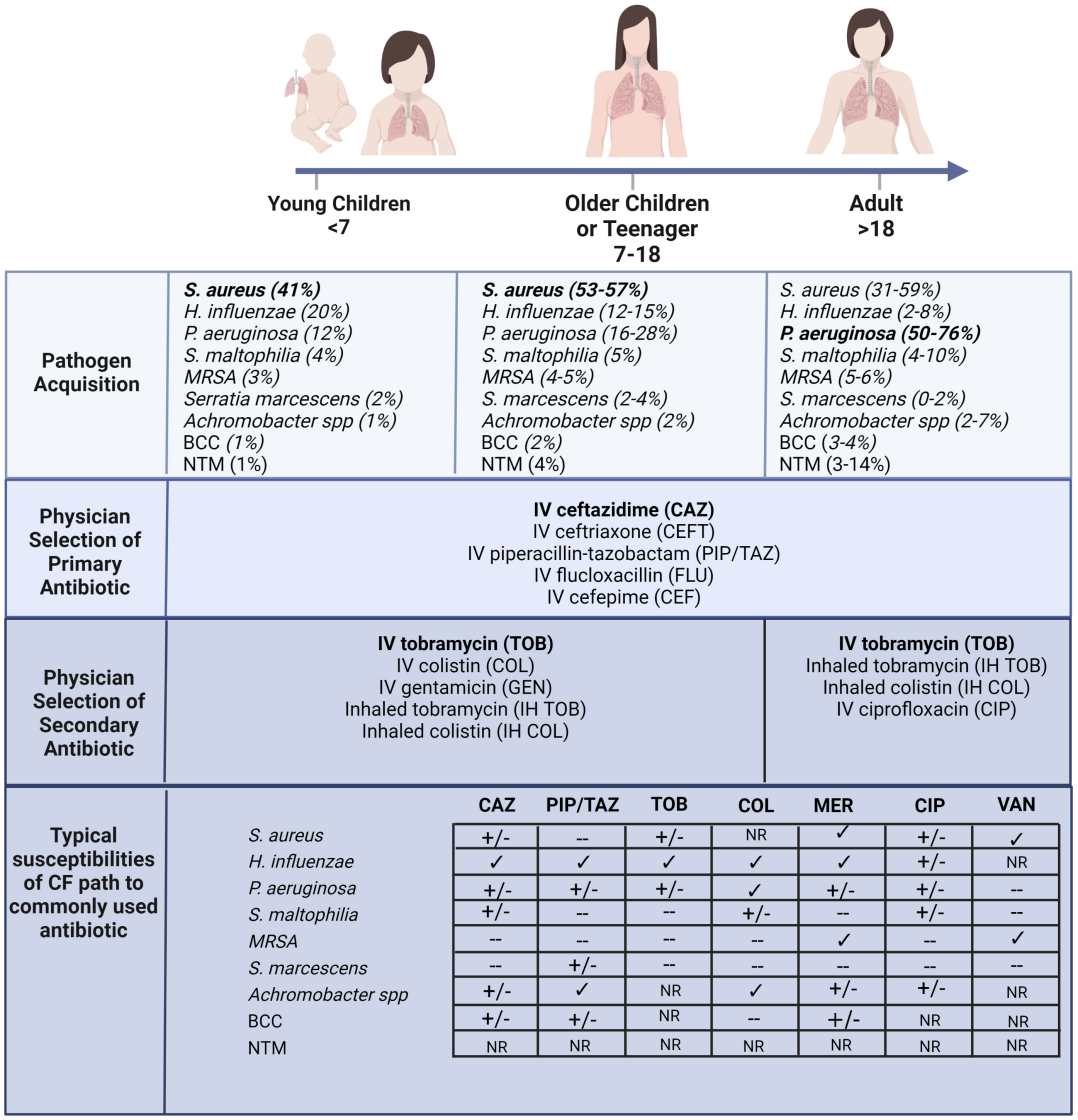
According to the Australian Cystic Fibrosis Data Registry 2020 (ACFDR 2020) (64), common microorganisms found in the respiratory tracts of individuals with CF, including the most common pathogen in early childhood *S. aureus*, and the most common pathogens in adulthood *P. aeruginosa*. Currie and colleagues reported the selection of intravenous primary and secondary inhaled or intravenous antibiotics by pediatrics or adult physicians across multiple CF centers in Australia (52). The susceptibility of a few common antibiotics (CAZ = ceftazimide; PIP/TAZ = piperacillin-tazobactam; TOB = tobramycin; COL = colistin; MER = meropenem; CIP = ciprofloxacin; VAN = vancomycin) of the common pathogens recovered from CF lung (60–63). √ indicates susceptibility, +/− indicates partial susceptibility, − indicates resistant and NR indicates not reported. For example, *S. aureus* typically exhibits multi-drug resistance, across most antibiotics including macrolides (55, 65). Furthermore, the MRSA strain confers resistance to β-lactams, quinolones and aminoglycosides (55, 65). The prominent CF pathogen, *P. aeruginosa*, also is developing broad spectrum resistance and other less common pathogens, including BCC, are resistant to most of the β-lactams, aminoglycosides, and cationic antimicrobial peptides (66). Created with BioRender.com.

A hallmark of CF lung disease includes hyper-inflammatory responses to early-life colonization and infection events that continue over life to develop into chronic inflammation (33–35). The acquisition of bacterial pathogens in CF airways appears to be an age-dependent sequence (Figure 1) (36). Bacterial-induced pulmonary exacerbations in infants and children with CF are often associated with *Haemophilus influenzae*, *Staphylococcus aureus*, and *Streptococcus pneumoniae* (37). As disease progresses with age, the CF airway becomes more susceptible to gram-negative bacteria, particularly *Pseudomonas aeruginosa*, which is highly associated with chronic airway inflammation (Figure 1) (38–41). In adults with CF, most pulmonary exacerbations are independent of new bacterial strain acquisition (38) or increases in the airway density of *P. aeruginosa* (40). Other less common pathogens include *Streptococcus pneumoniae*, *Stenotrophomonas maltophilia*, *Moraxella catarrhalis*, *methicillin-resistant Staphylococcus aureus* (MRSA), *Burkholderia cepacia complex* (BCC), *Achromobacter xylosoxidans*, and nontuberculous mycobacteria (NTM) (42–46). Selection criteria, including the delivery method (e.g., intravenous or inhalation) and treatment duration, also influence which type of antibiotic could be chosen for CF pulmonary infections. For example, inhalation therapy is advantageous since it can directly target the lower respiratory tract and site of infection with higher doses of antibiotics and exhibits reduced systemic toxicity and side effects (32, 47). Administration of inhaled antibiotics, including tobramycin, colistin, aztreonam lysine and levofloxacin are now commonly used to manage infections in CF (48–50) and others, such as ceftaroline and vancomycin, have been assessed for their ability to eradicate specific infections including MRSA (51). In Australia, CF physicians typically select inhaled tobramycin or colistin as a secondary antibiotic in combination with a primary intravenous antibiotic such as ceftazidime (Figure 1) (52). Although prolonged use of inhaled antibiotics has not been reported for newly acquired multidrug-resistant bacterial strains, the long-term clinical impacts of high-dose antibiotics on the perturbation of airway microbes are still unknown. Antibiotic susceptibility profiles of various CF pathogens are also summarized in Figure 1 (53–58). The extensive consumption of antibiotics has resulted in increased isolated MDR pathogens. Indeed, polymicrobial interaction between *P. aeruginosa* and *S. aureus* has resulted in tobramycin resistance in isolates recovered from children with CF (59). The development of resistance towards the last-line option, colistin, in isolates derived from individuals with CF is particularly concerning (60–63).

### Alternative treatments and phage therapy

Antibiotic resistance has become a global issue, and the prescription of antibiotics by physicians has increased by 10 times in response to the expectation of patients (67). Long-term use of antibiotics and dosing of these drugs have become a major concern in CF, leading to the emergence of resistance over time. Alternative treatment methods have been and are currently being explored to address this, including targeting bacterial virulence and resistance (66). Anti-virulence compounds such as quorum sensing inhibitors (67, 68) and iron chelation (71, 72) successfully prevented bacterial aggregate, inhibited biofilm formation, reduced pathogenicity, and increased susceptibility to traditional antimicrobials. Strategies targeting resistance have included investigating efflux pump inhibitors (73, 74), anti-sense oligomers (75, 76), immunotherapy (77), host defence peptides (78, 79) and bacteriophages (4). Many of these strategies are still in the exploration and validation phases and are years off from translating to clinical care practice. The vital need for swift translation of alternative therapy into clinical use has identified phage therapy as the top candidate due to its successful use in humans when approved on compassionate grounds.

Phage therapy is highly applicable in CF, where most healthcare costs are associated with recurrent hospital visits due to chronic bacterial infections (80). It holds many advantages over conventional antibiotic treatments used in CF, including shorter treatment periods (81–83), bacterial specificity (84), potent efficacy (85) and low toxicity (86). In the last 5 years, phage therapy has successfully been used to treat more than 30 individuals with CF (Supplementary Table S1) against various infections, including *P. aeruginosa*, *S. aureus*, *B. dolosa*, and *M. abscessus*. Phages were used singularly or in combination with other phages, typically called phage cocktails (Supplementary Table S1). Various treatment administration routes were also observed, including intravenous injection, oral or inhalation. Of significance were the reported improved clinical benefits (including a reduction in sputum and cough and improved lung function) but, more importantly, infection eradication. Furthermore, phage therapy was well tolerated, with no adverse events reported. Of these cases, the largest involved 20 patients (primarily individuals with CF), where mycobacterial infections were successfully treated with phage (87). Patients were well-tolerated with treatment, and phage resistance was not observed (87). Recently, a multi-center clinical trial was successfully conducted assessing the safety, tolerability, pharmacokinetics, and pharmacodynamics of a multi-phage candidate in people with CF suffering chronic *P. aeruginosa* pulmonary infections (88). Encouragingly, results showed that the formulation was well tolerated, was effectively delivered to the site of infection, and reduced bacterial load in participants (88).

## Airway mucus and CF

Normal airway mucus is a soft hydrogel composed of 90-95% water, mucus lipids and mucus proteins (such as glycoproteins) and ~1–5% high-molecular-weight mucins (89). Mucus forms a protective layer above the airway epithelium to trap airborne particles, including pathogens. Under normal circumstances, cell surface glycosylation is attributed to the gene expression of glycosyltransferases, as these enzymes are involved in the biosynthesis of glycan products (90, 91). The O-linked glycosylation is when glycans (carbohydrates/oligosaccharides) are added to mucins. A previous study by our group has demonstrated distinct mucins and glycosyltransferase profiles before and after rhinovirus infection (92). Not only did the expression of mucins vary between primary cells from CF and non-CF individuals, but the glycosyltransferases which form the eight core types of mucin O-glycans were also differentially expressed. These glycosyltransferases, including galactose, N-acetylglucosamine, fucose and terminal sialic acid or sulphate, form the various glycan moieties on the mucosal surface (93). The dysregulated mucin production suggests that other regulatory mechanisms, such as appropriate mucin packaging and secretion, might compromise CF cells (92). These variations are also linked to increased bacterial infection and inflammation due to the altered biomolecular properties of CF mucus (17, 94). Whether applying phage therapy to treat chronic infection in CF could further alter the glycosyltransferases profile of CF mucus requires a thorough understanding of the phage behavior and efficacy in the CF airway.

Before clinical translation, safety and efficacy assessment *in vivo* needs to be addressed, and small animal models have been typically used in this setting (95, 96). A systematic review assessing phage efficacy in various *in vivo* infection models showed significantly improved survival with treatment and reduced bacterial tissue burden (97). In addition, animal models have been used to effectively assess phage-antibiotic interactions (98) and the development of phage resistance (99). Furthermore, when combined with mathematical modelling, they have been used to quantify the dose and route of phage administration to fully capture phage efficacy, bacterial kinetics, and animal outcomes. The authors suggested that when accounting for host immune responses (mucins, cytokine, immune cells), this model could characterize the synergism between phages and the host innate immune system on bacteria elimination (100). In the setting of CF, animal models, including βENaC-Tg mice, CF pigs, CFTR rats, and ferrets, have all shed light on the early onset and progression of lung disease (101–105). However, these models have several limitations, including that CF pigs lack effective host defence mechanisms against bacterial pathogens. The CF ferrets model develop severe CF-like lung disease rapidly despite antibiotic prophylaxis and exhibits spontaneous lung infection which requires ongoing high levels of care (102, 103). With no single animal model completely recapitulating progressive CF lung disease, assessing phage efficacy in this setting may prove challenging (106).

One alternative is (107) primary airway epithelial cells obtained from paediatric CF airways that can be established *in vitro* and subsequently infected with pathogens and/or phages. Work conducted has shown little impact on to host airway, with no viability loss or significant inflammatory responses to phage reported (108). However, primary monolayer cultures do not fully reflect the CF mucosal epithelium. In order to more accurately recapitulate the *in vivo* CF airway, a more sophisticated proxy should be employed. Specifically, airway cells established at air-liquid interface (ALI) undergo differentiation and polarization, forming a mixed population of epithelial cells (ciliated, basal, goblet cells) to mimic the *in vivo* condition (109, 110). Importantly, since these models also contain goblet cells that produce mucus, it enables researchers to investigate the interplay between phage, mucus and pathogens and how the unique mucus properties seen in CF affect these relationships.

## The interplay between CF mucus, pathogens and phage

The defective airway physiology of CF impairs mucociliary clearance, triggers thick, dense mucus production, promotes the establishment of microbes and affects host immune responses to infection and inflammation. In these environments, including the CF airway, bacteria live in aggregates forming clusters of communities suspended within the airway mucus and its self-secreted protein, called biofilms (53, 111). Although an ideal phage formulation to treat CF lung infection and penetrate biofilms is yet to be identified, phage cocktails targeting different receptors have effectively reduced the emergence of bacteriophage insensitive mutants (BIMs) (112). Others have also shown that concomitant treatment of phages and antibiotics (also termed phage-antibiotic synergy; PAS) results in synergistic efficacy and reduced antibiotic resistance (56, 57, 113). Several case studies have reported favorable outcomes of phage-antibiotic treatments in various scenarios (13, 14, 98, 114–116) Specifically and pertinent to this review, an adult with CF suffering a multi-drug resistance *P. aeruginosa* infection was successfully treated with a combination of a multi-phage cocktail and ciprofloxacin andpiperacillin–tazobactam (14).

Despite this, phage activity is often limited by biofilm formation due to the impermeability of the biofilm matrix, preventing phages from reaching their receptors on the bacteria cell membrane. However, certain phages encode capsular depolymerase, which binds and breaks down the polysaccharide layer of bacteria for binding of phages to the receptors on bacteria surface (117) or degradation of exopolysaccharides which enable biofilm penetration, and hence is subsequently bactericidal (117–119). However, the effects of CF mucus on phage-bacteria/biofilm activity in the CF airway are still largely unknown and require investigation.

In response to phage, several bacterial defence mechanisms are activated, including modification of surface receptors (120, 121), prevention of phage DNA injection into bacteria cells (122), cleavage of phage DNA (123, 124), and finally, suicidal induction of infected bacterial cells (125, 126). However, whether CF mucus facilitates phage resistance while treating a chronic bacterial infection in CF lung is currently unknown. A recent study has provided some insight into this (130). Specifically, the authors demonstrated that the co-existence of a fish pathogen *Flavobacterium columnare* and their virulent phage in the presence of mucin lead to a significant increase in phage resistance, particularly CRISPR-Cas (an adaptive immune system that recognizes phage genomes) (127). The presence of static CF mucus flakes in the human lung creates a low-oxygen environment with an accompanying anaerobic layer (128). Although this may facilitate bacterial attachment, it could stimulate biofilm growth with the generation of dormant persister cells in its deeper layers, inhibiting phage propagation since nutrient resources are scarce.

## The potential impact of CF mucus on phage therapy

**Figure 2:**
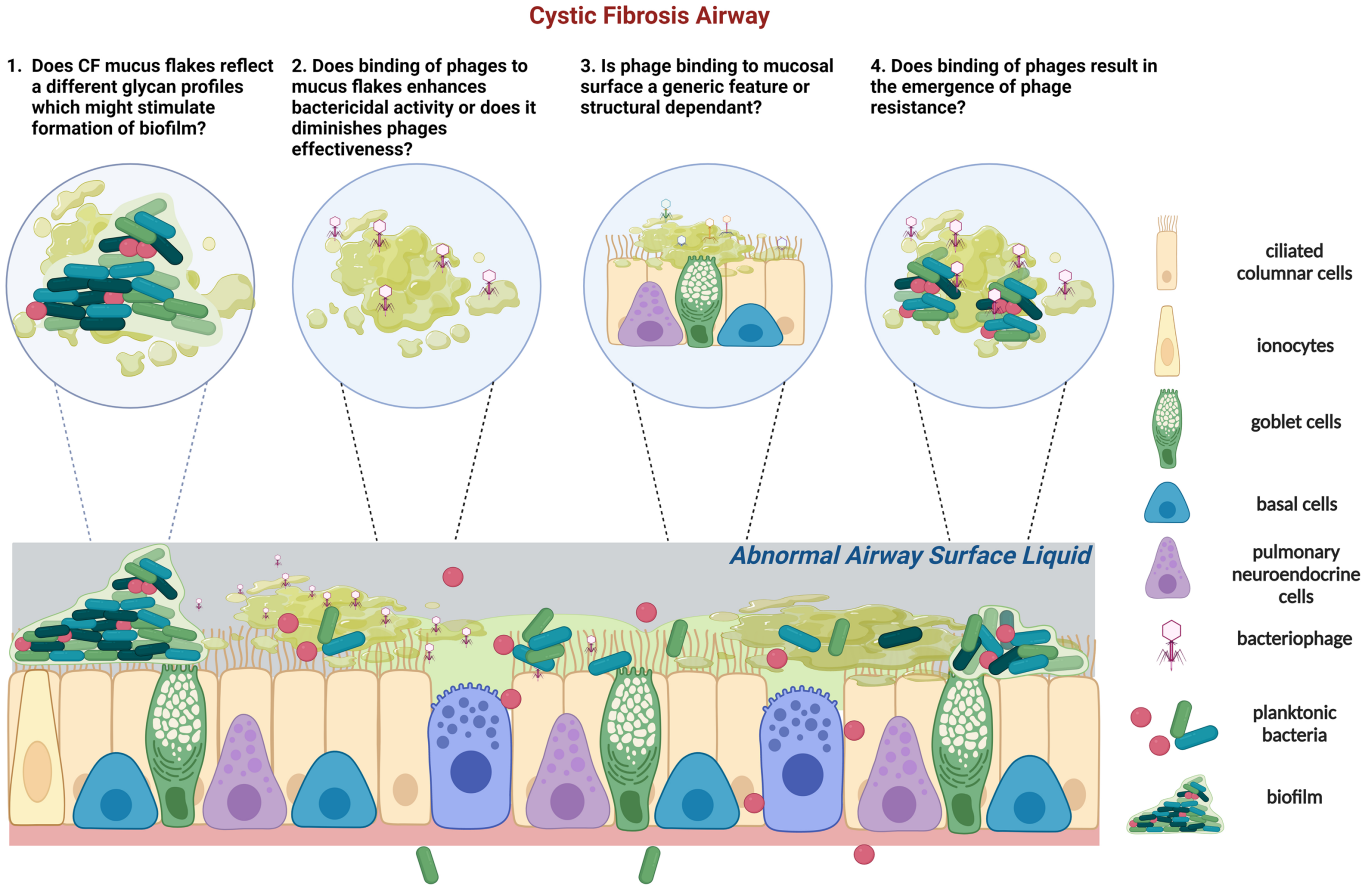
Schematic representation of the tripartite (phage-bacteria-epithelial cells) of CF epithelium is characterized by low airway surface liquid and thick and dense mucus containing mucus flakes and hyperconcentrated mucins. In CF, commensal bacteria and phage communities are dysregulated following long-term pathogenic chronic bacterial infection with dominant species and limited phages. (1) In addition, glycans that form mucins can also regulate the adhesion of *P. aeruginosa*. It is unknown if CF mucus flakes constitute a different glycan profile which could stimulate biofilm formation. (2) Phages can adhere to mucin directly; it is unknown how or if phages bind to CF mucus flakes directly and if their capsid proteins are subseqeuntly altered. Adherence of phage to CF mucus may facilitate phage enrichment and persistence within the microenvironment, resulting in a potent phage phenotype or vice versa. (3) Previous studies identified that certain phages, but not all, bind to mucins and diminish bacterial killing activity. Of concern, it is unknown if the binding activity of phages to mucin is a generic feature or size and structure dependent. (4) Importantly, if CF mucus results in the emergence of phage resistance which contradicts the theory of virulence enhancement by mucus, other therapeutic strategies might need to be applied in conjunction with the proposed phage therapy in CF. Created with BioRender.com.

The type of phages within CF lungs appears more similar, mainly derived from the pathogens that persist in the CF airways for longer, compared to a more diverse phage population in healthy airways (129) (Figure 2). However, very limited studies have reported the phage-mucus interactions in the airway and the potential impacts of phage efficacy on respiratory bacterial infection. Recent evidence suggests that phages can bind to aspects of normal mucus that improve their bactericidal activity (11). However, little is known about how the CF mucus impacts phage functionality (Figure 2). Phage glycan-binding proteins bind to the bacterial surface for infection, while glycan ligands on the surrounding environment, including those on mucins, are modified to promote phage retention in the gastrointestinal tracts (130). Barr et al. (131) have demonstrated the binding of a coliphage to mucin through the Ig-like domains in its capsid proteins called T4 Hoc protein (131). This binding increased the retention of T4 in the environment, allowing more interaction between phage and *E. coli* and serving as a critical regulator for phage-mediated bacterial lysis (132). Other work in non-respiratory research has shown that phages adhering to mucin have enhanced virulence toward bacteria (11, 133), empowering the chances of phage encountering bacterial hosts and providing additional mucosal immunity protection. Almeida et al. (133) demonstrated using a natural infection system that tailed phages with Ig-like domains in the phage capsids preferentially bind to mucin-containing agar. Phage concentration was previously found to be 4.4-fold higher within the mucus layer and associated with phage enrichment (134). Phages adhere to mucus glycans through weak binding interactions with the capsid proteins (127). This binding mechanism enables the subdiffusive motion of phages within mucosal surfaces, providing notably enhanced encounter rates with bacterial hosts (131, 135). These benefits allow mucus-adherent phage to propagate throughout the mucus layer, forming a non-host-derived layer of immunity. In addition, the direct evolutionary benefits of phage binding to the mucosal surface are the increased cost of bacterial virulence and modifications of bacterial phenotype to be more susceptible to phage infections in *Flavobacterium columnare* and *Aeromonas* sp. (133).

In contrast, Green et al. (136) found that incubating certain phages with porcine intestinal mucins reduced or inhibited bacterial killing. The bactericidal activity was restored after adding the mucolytic agent N-acetyl cysteine (NAC). Nevertheless, the same study identified a novel phage ES17, whose bactericidal activity was enhanced by binding to human heparan sulfated proteoglycans in mucus, forming a protective layer on the intestinal epithelium (136). In the CF airway, it is unknown how or if phages bind to CF mucus or the airway glycans directly and if phage binding enhances the bactericidal activity and virulence or vice versa, as observed in the gut lines previously.

In addition to regulating phages, glycans that form mucins can also regulate the physiology *P. aeruginosa*, including virulence and adhesion (137). A recent *in vitro* study found that *P. aeruginosa* induces contractions of luminal mucus, which accelerates bacterial aggregation and biofilm formation. This study showed that the host mucus production protects epithelium from acute virulence yet provides a breeding ground for biofilm and chronic infections (138). In the scenario of CF, dysregulated mucin production may enhance biofilm formation on the mucosal surface, however, it may counteract the positive interaction of mucus and phages on bactericidal activity. Future research addressing whether mucus increases or decreases the chance of phages and bacterial interaction, virulence, and enhancing phage resistance within the mucus layers may be key to successful phage therapy in CF.

The mucin content assessment in BALf of young children with CF has shown both elevated mucin concentration and the presence of mucus flakes evident very early in life (17). Mucus and mucin polymers, including MUC5AC, MUC2 and MUC5B, have been found to substantially diminish the activity of polymyxin and fluoroquinolone antibiotics against *P. aeruginosa* (139). However, what remains unknown is whether phages bind to CF mucus directly and if their capsid proteins are resultingly altered. The consideration here is whether this mucus will alter the glycan residues on mucin, affecting bacteria binding and modifying the efficacy of phage therapy. Would it be possible for this inhibition to also allow for increased predation as the bacteria move within the mucin microenvironment? Future work should assess if CF mucus can create an antimicrobial layer that reduces bacterial attachment and lessens epithelial cell death, as observed in a gut cell model (11). Furthermore, investigations are warranted to investigate whether phages retained in the CF mucus layer facilitate phage enrichment and persistence within the microenvironment, resulting in a potent phage phenotype or vice versa.

## Conclusion

In summary, AMR in CF has long-term clinical consequences, and the hyper-concentrated mucus with a dysfunctional structure strikingly impacts CF airways, providing the right environment for chronic bacterial infections. With little investment in discovering new antibiotics, assessing the implementation of phage therapy as an alternative therapeutic strategy for AMR pulmonary infections is critical. This may include using 3D airway cultures to examine phage tropism for CF pathogens and determine the impact of phage therapy on bacterial biofilm penetration. In addition, research is needed to elucidate the interactive relationships between phage, CF pathogens and the host airway epithelium, including impacts of the dehydrated mucus typical of the CF airway. Furthermore, a recent study suggests that phage may be able to infect a much broader repertoire of bacteria beyond a single species (140). This raises the question of whether they also cause microbiome dysbiosis in the lung by infecting the resident commensal population. All these necessary research pieces must be conducted to understand the translational implications of such a therapy in CF. Many unanswered questions include determining the best administration route, concomitant use of antibiotics or mucolytic agents, length of the treatment period, and phage formulation. Furthermore, additional basic research is needed to predict these parameters and accurately measure host immune responses.

## Author contributions

K-ML, SS, and AK conceptualized the contents of the manuscript, critically reviewed, and edited the manuscript. K-ML wrote the first draft of the manuscript. All authors contributed to the article and approved the submitted version.

## Funding

K-ML is a Conquer Cystic Fibrosis Research Fellow and supported by a Vertex Innovation Mentored Award. SS holds an NHMRC Investigator Grant (#2007725) and AK is a Rothwell Family Fellow.

## Conflict of interest

The authors declare that the review was conducted in the absence of any commercial or financial relationships that could be construed as a potential conflict of interest.

## Publisher’s note

All claims expressed in this article are solely those of the authors and do not necessarily represent those of their affiliated organizations, or those of the publisher, the editors and the reviewers. Any product that may be evaluated in this article, or claim that may be made by its manufacturer, is not guaranteed or endorsed by the publisher.
