# Supplementary material for: Pulmonary bacteriophage and cystic fibrosis airway mucus: friends or foes?
Source: Front Med (Lausanne). 2023 May 17;10:1088494. doi: 10.3389/fmed.2023.1088494 (PMC10230084; doi:10.3389/fmed.2023.1088494)
Supplement: Supplementary file 1 [file Data_Sheet_1.pdf]

Table 1 Case studies treating CF pulmonary infection using phage therapy.

Year	Patient Population (Age years; Sex)	Country	Infection Pathogen	Phage Preparation	Administration Route	Dosage and Regimen	Concomitant Treatment	Reported Adverse Events	Efficacy and Outcome		Reference
									Clinical Improvement	Bactericidal Eradication	
2011	1 patient (7, F)		*Staphylococcus aureus*	Single Phage	Inhalation	Multidose	Antibiotics	No	Yes	Yes	(Kvachadze et al., 2011)
2018	1 patient (17, F)	Georgia	MDR *Achromobacter xylosoxidans*	Phage Cocktail	Oral and Inhalation	Multi-dose	Antibiotics	Not recorded	Yes	Not Recorded	(Hoyle et al., 2018)
2019	4 patients (16-38, F)	USA	MDR *Pseudomonas aeruginosa*	Single Phage	Inhalation	Multi-dose	No	No	Not Recorded	Yes	(Koff et al., 2019)
2019	1 patient (28, F)	USA	MDR *Burkholderia dolosa*	Single Phage	IV	Multi-dose	Antibiotics	No	Yes	Partial	(Aslam et al., 2019)
2019	1 patient (15, F)	USA	MDR *Mycobacterium abscessus*	Phage Cocktail	IV	Multi-dose	Antibiotics	No	Yes, but relapsed after 2 months	Yes, but increased count after 2 months	(Dedrick et al., 2019)
2019	1 patient (26, F)	USA	MDR *Pseudomonas aeruginosa*	Phage Cocktail	IV	Multi-dose	Antibiotics	No	Yes	No	(Law et al., 2019)
2020	4 patients (Not Recorded)	USA	MDR *Pseudomonas aeruginosa*	Single Phage	Inhalation	Multi-dose	No	No	Yes	Yes	(Stanley et al., 2020)
2020	1 patient (10, F)	USA	PDR *Achromobacter spp*	Single Phage	IV	Multi-dose	Antibiotics	No	Yes	Yes	(Gainey et al., 2020)

2021	1 patient (12, M)	France	PDR *Achromobacter xylosoxidans*	Phage Cocktail	Oral and Inhalation	Multi-dose	Antibiotics	No	Yes	Yes	(Lebeaux et al., 2021)
2021	1 patient (43, M)	Georgia	MDR *Pseudomonas aeruginosa*	Phage Cocktail	Oral and Inhalation	Multi-dose	Antibiotics	No	Yes	Partial	(Zaldastanishvili et al., 2021)
2022	1 patient (26, M)	USA	MDR *Mycobacterium abscessus*	Phage Cocktail	IV	Multi-dose	Antibiotics	No	Yes	Yes	(Nick et al., 2022)
2022	20 patients (>5, Not Recorded)	USA	MDR *Mycobacterium spp*	Single Phage/Phage Cocktail	IV (Inhalation in 2 patients	Multi-dose	Antibiotics	No	Yes		(Dedrick et al., 2023)

ICU, intensive care unit; IV, intravenous; MDR, multi-drug resistant; PDR, pan drug resistant, *spp,* species.

References:

Aslam, S., Courtwright, A. M., Koval, C., Lehman, S. M., Morales, S., Furr, C. L. L., Rosas, F., Brownstein, M. J., Fackler, J. R., Sisson, B. M., Biswas, B., Henry, M., Luu, T., Bivens, B. N., Hamilton, T., Duplessis, C., Logan, C., Law, N., Yung, G., … Schooley, R. T. (2019). Early clinical experience of bacteriophage therapy in 3 lung transplant recipients. *American Journal of Transplantation*, *19*(9), 2631–2639. https://doi.org/10.1111/ajt.15503

Dedrick, R. M., Guerrero-Bustamante, C. A., Garlena, R. A., Russell, D. A., Ford, K., Harris, K., Gilmour, K. C., Soothill, J., Jacobs-Sera, D., Schooley, R. T., Hatfull, G. F., & Spencer, H. (2019). Engineered bacteriophages for treatment of a patient with a disseminated drug-resistant Mycobacterium abscessus. *Nature Medicine 2019 25:5*, *25*(5), 730–733. https://doi.org/10.1038/s41591-019-0437-z

Dedrick, R. M., Smith, B. E., Cristinziano, M., Freeman, K. G., Jacobs-Sera, D., Belessis, Y., Whitney Brown, A., Cohen, K. A., Davidson, R. M., van Duin, D., Gainey, A., Garcia, C. B., Robert George, C. R., Haidar, G., Ip, W., Iredell, J., Khatami, A., Little, J. S., Malmivaara, K., … Hatfull, G. F. (2023). Phage Therapy of Mycobacterium Infections: Compassionate Use of Phages in 20 Patients With Drug-Resistant Mycobacterial Disease. *Clinical Infectious Diseases*, *76*(1), 103–112. https://doi.org/10.1093/cid/ciac453

Gainey, A. B., Burch, A., Brownstein, M. J., Brown, D. E., Fackler, J., Horne, B., Biswas, B., Bivens, B. N., Malagon, F., & Daniels, R. (2020). Combining bacteriophages with cefiderocol and meropenem/vaborbactam to treat a pan-drug resistant *Achromobacter* species infection in a pediatric cystic fibrosis patient. *Pediatric Pulmonology*, *55*(11), 2990–2994. https://doi.org/10.1002/ppul.24945

Hoyle, N., Zhvaniya, P., Balarjishvili, N., Bolkvadze, D., Nadareishvili, L., Nizharadze, D., Wittmann, J., Rohde, C., & Kutateladze, M. (2018). Phage therapy against Achromobacter xylosoxidans lung infection in a patient with cystic fibrosis: a case report. *Research in Microbiology*, *169*(9), 540–542. https://doi.org/10.1016/j.resmic.2018.05.001

Koff, J. L., Chan, B. K., Stanley, G. L., Geer, J. H., Grun, C., Kazmerciak, B., & Turner, P. E. (2019). Clinical Use of Inhaled Bacteriophages to Treat Multi-Drug Resistant Pseudomonas Aeruginosa. *B109. What's new with clinical lung infections and pneumonia?*, A7381–A7381. https://doi.org/10.1164/ajrccm-conference.2019.199.1_MeetingAbstracts.A7381

Kvachadze, L., Balarjishvili, N., Meskhi, T., Tevdoradze, E., Skhirtladze, N., Pataridze, T., Adamia, R., Topuria, T., Kutter, E., Rohde, C., & Kutateladze, M. (2011). Evaluation of lytic activity of staphylococcal bacteriophage Sb-1 against freshly isolated clinical pathogens. *Microbial Biotechnology*, *4*(5), 643–650. https://doi.org/10.1111/j.1751-7915.2011.00259.x

Law, N., Logan, C., Yung, G., Furr, C. L. L., Lehman, S. M., Morales, S., Rosas, F., Gaidamaka, A., Bilinsky, I., Grint, P., Schooley, R. T., & Aslam, S. (2019). Successful adjunctive use of bacteriophage therapy for treatment of multidrug-resistant Pseudomonas aeruginosa infection in a cystic fibrosis patient. *Infection 2019 47:4*, *47*(4), 665–668. https://doi.org/10.1007/S15010-019-01319-0

Lebeaux, D., Merabishvili, M., Caudron, E., Lannoy, D., Van Simaey, L., Duyvejonck, H., Guillemain, R., Thumerelle, C., Podglajen, I., Compain, F., Kassis, N., Mainardi, J.-L., Wittmann, J., Rohde, C., Pirnay, J.-P., Dufour, N., Vermeulen, S., Gansemans, Y., Van Nieuwerburgh, F., & Vaneechoutte, M. (2021). A Case of Phage Therapy against Pandrug-Resistant Achromobacter xylosoxidans in a 12-Year-Old Lung-Transplanted Cystic Fibrosis Patient. *Viruses*, *13*(1), 60. https://doi.org/10.3390/v13010060

Nick, J. A., Dedrick, R. M., Gray, A. L., Vladar, E. K., Smith, B. E., Freeman, K. G., Malcolm, K. C., Epperson, L. E., Hasan, N. A., Hendrix, J., Callahan, K., Walton, K., Vestal, B., Wheeler, E., Rysavy, N. M., Poch, K., Caceres, S., Lovell, V. K., Hisert, K. B., … Davidson, R. M. (2022). Host and pathogen response to bacteriophage engineered against Mycobacterium abscessus lung infection. *Cell*, *185*(11), 1860-1874.e12. https://doi.org/10.1016/j.cell.2022.04.024

Stanley, G. L., Chan, B., Ott, I., Mayo, E., Harris, Z. M., Sun, Y., Hu, B., Rajagopalan, G., Turner, P., & Koff, J. L. (2020). Bacteriophage Therapy Decreases Pseudomonas Aeruginosa Lung Inflammation. *B29. Infection and immune interplay in lung injury*, a2977–a2977. https://doi.org/10.1164/ajrccm-conference.2020.201.1_MeetingAbstracts.A2977

Zaldastanishvili, E., Leshkasheli, L., Dadiani, M., Nadareishvili, L., Askilashvili, L., Kvatadze, N., Goderdzishvili, M., Kutateladze, M., & Balarjishvili, N. (2021). Phage Therapy Experience at the Eliava Phage Therapy Center: Three Cases of Bacterial Persistence. *Viruses*, *13*(10), 1901. https://doi.org/10.3390/v13101901
